# Identification of the Hub Genes Involved in Chikungunya Viral Infection

**DOI:** 10.7759/cureus.57603

**Published:** 2024-04-04

**Authors:** Sanaa Ahmed, Ahmed Salem, Nema Hamadan, Maha Khalfallah, Mohamed Alfaki

**Affiliations:** 1 Pharmacology, Faculty of Pharmacy, University of Khartoum, Khartoum, SDN; 2 Biological and Biochemical Sciences, Faculty of Chemical Technology, University of Pardubice, Pardubice, CZE; 3 Histopathology and Cytology, University of Ibn Sina, Khartoum, SDN; 4 Zoology, Faculty of Science, University of Khartoum, Khartoum, SDN; 5 Research, Sidra Medicine, Doha, QAT

**Keywords:** hub genes, cellular signaling pathways, protein-protein interaction (ppi) network, differentially expressed genes (degs), chikungunya virus (chikv)

## Abstract

Background

Chikungunya virus (CHIKV) infection poses a significant global health threat, necessitating a deeper understanding of its molecular mechanisms for effective management and treatment. This study aimed to understand the molecular and genetic mechanisms of CHIKV infection by analyzing microarray expression data.

Methodology

National Center for Biotechnology Information (NCBI) GEO2R with an adjusted p-value cut-off of <0.05 and |log_2_FC ≥ 1.5| was used to identify the differentially expressed genes involved in CHIKV infection using microarray data from the Gene Expression Omnibus (GEO) database, followed by enrichment analysis, protein-protein interaction (PPI) network construction, and, finally, hub gene identification.

Results

Analysis of the microarray dataset revealed 25 highly significant differentially expressed genes (DEGs), including 21 upregulated and four downregulated genes. PPI network analysis elucidated interactions among these DEGs, with hub genes such as *ACTB* and *CTNNB1* exhibiting central roles. Enrichment analysis identified crucial pathways, including leukocyte transendothelial migration, regulation of actin cytoskeleton, and thyroid hormone signaling, implicating their involvement in CHIKV infection. Furthermore, the study highlights potential therapeutic targets such as *ACTB *and *CTNNB1*, which showed significant upregulation in infected cells.

Conclusions

These findings underscore the complex interplay between viral infection and host cellular processes, shedding light on novel avenues for diagnostic marker discovery and advancing antiviral strategies. In this study, we shed light on the molecular and genetic mechanisms of CHIKV infection and the potential role of *ACTB *and *CTNNB1* genes.

## Introduction

Chikungunya virus (CHIKV) is a single-stranded, positive-sense, RNA virus that belongs to the Togaviridae family and the Semliki Forest virus antigenic complex. It is classified as an alphavirus and is primarily transmitted by *Aedes aegypti* and *Aedes albopictus* mosquitoes. The virus was first identified in Africa during an outbreak of a febrile illness [1]. Since then, it has spread to over 40 countries, causing significant health concerns due to its ability to cause acute and chronic musculoskeletal pain, particularly in vulnerable populations during outbreaks [2]. The virus caused urban outbreaks in Thailand in 1967 and India in the 1970s [3]. According to the World Health Organization reports, it has now been identified in over 110 countries across the globe, including Asia, Africa, Europe, and the Americas.

CHIKV infection is a global health concern that requires further investigation to understand its long-term consequences, disease pathogenesis, and epidemiological implications. This knowledge is essential for developing effective preventive and therapeutic interventions. Research should focus on understanding the virus’s transmission dynamics, particularly the roles of different mosquito species and alternative modes of transmission. CHIKV infection causes fever, headache, rash, muscle pain, and severe joint pain. Currently, no specific antiviral treatments are available for CHIKV infection, and patients are managed during the acute phase of infection with pharmacological interventions, such as dipyrone or paracetamol, based on pain intensity evaluated using the visual analog scale (VAS). Although most infections tend to resolve independently, and clinicians take necessary precautions, including assessing patient allergies and comorbidities to prevent imprecise prescriptions [4], there are increasing reports confirming that CHIKV infection is associated with increased risk of death for up to three months following the infection due to ischemic heart conditions, cerebrovascular disorders, and diabetes, as the infection has been shown to exacerbate underlying diseases, potentially leading to serious outcomes such as stroke, acute myocardial infarction, kidney manifestations, and neurological disorders [5].

This study aimed to improve the prognosis of CHIKV infection through integrative transcriptomic-bioinformatics analysis to identify differentially expressed genes (DEGs), assess protein-protein interaction (PPI) networks, and leverage microarray and Gene Expression Omnibus (GEO) databases. In addition, it aims to target the potential therapeutic value using hub gene and cluster identification methods.

## Materials and methods

Microarray data and GEO database

The GEO (https://www.ncbi.nlm.nih.gov/gds/) is a publicly accessible and operational genomics repository that houses extensive data related to gene expression, including high-throughput information from gene expression experiments, DNA microarray chips, and other related datasets. Using the keywords “homo sapiens,” “Chikungunya infection,” and “Expression by array” in this investigation, the microarray data from GSE49985 [6] was examined. The analysis was conducted using the GPL15207 Affymetrix Human Gene Expression Array platform. The study represented expression profiling in HEK293t cells infected with CHIKV where the experiment included growing cells in six well plates in which two cell plates were considered as control whose RNA isolates were detected at time zero-hour infected Rep1 and zero-hour infected Rep 2, and four plates were the study samples in which two RNA expression isolates were collected at 12 as 12-hour infected Rep 1 and 12-hour infected Rep 2 and the other two were collected at 24 hours following the infection as 24-hour Rep1 and 24-hour Rep2.

Identification of DEGs

To identify DEGs between the samples and control, the GEO2R tool (https://www.ncbi.nlm.nih.gov/geo/geo2r/) [7] was employed. GEO2R tool is an online interactive network application on the National Center for Biotechnology Information (NCBI) that uses the R package limma and GEO query to enable users to compare two or more sets of samples within a GEO dataset, with the default setting of statistical significance threshold as p < 0.05 and |log2FC ≥ 2|. Then, the CHIKV infection biomarkers were identified as highly significant DEGs where the adjusted p-value cut-off was <0.05 and the |log2FC ≥ 1.5|.

PPI network construction

The analysis of the PPI network was performed using the Search Tool for the Retrieval of Interacting Genes/Proteins (STRING) platform (http://string-db.org) [8], which is an online tool used to consolidate a vast array of known and predicted protein-protein association data, facilitating comprehensive assessments of these interactions as functional enrichment analysis. Subsequently, the obtained PPI network was visualized by the desktop Cytoscape software (https://cytoscape.org/) [9] utilizing the STRING application as a plugin tool.

Hub genes and cluster identification

The cytoHubba plugin within the Cytoscape software was utilized to identify the hub genes in the PPI network represented by nodes and edges. These hub genes were selected based on higher ranks in the analysis, indicating their significant connections and interactions within the PPI network. Then, these hub genes were filtered using a confidence (score) cutoff of 0.4 and ranked by maximal clique centrality (MCC). The top cluster genes involved in the PPI were obtained using the Molecular Complex Detection (MCODE) plugin on Cytoscape with a degree cutoff of 2, node density cutoff of 0.1, node score cutoff of 0.2, maximum depth of 100, and K-core of 2.

Enrichment analysis using the KEGG pathway for the hub genes

To investigate the relevant biological functions and signaling pathways of the hub genes the Enrichr/ Kyoto Encyclopedia of Genes and Genomes (KEGG) pathway analysis (https://maayanlab.cloud/Enrichr/) [10] was employed. This analysis is a comprehensive resource for curated gene sets and a powerful search engine that aggregates biological knowledge for further scientific discoveries. The KEGG pathway tool offers a platform for the qualitative interpretation of genomic sequences and various biological data, and it encompasses systematic, genomic, and chemical information, along with a dedicated category for human-specific health-related data. The outcomes of this analysis were then presented visually using the bioinformatics.cn tool (https://www.bioinformatics.com.cn/) [11], which was also used to construct a volcano plot for the hub genes to define the location of the hub genes within the DEGs in the dataset.

## Results

Microarray data and GEO database

**Figure 1:**
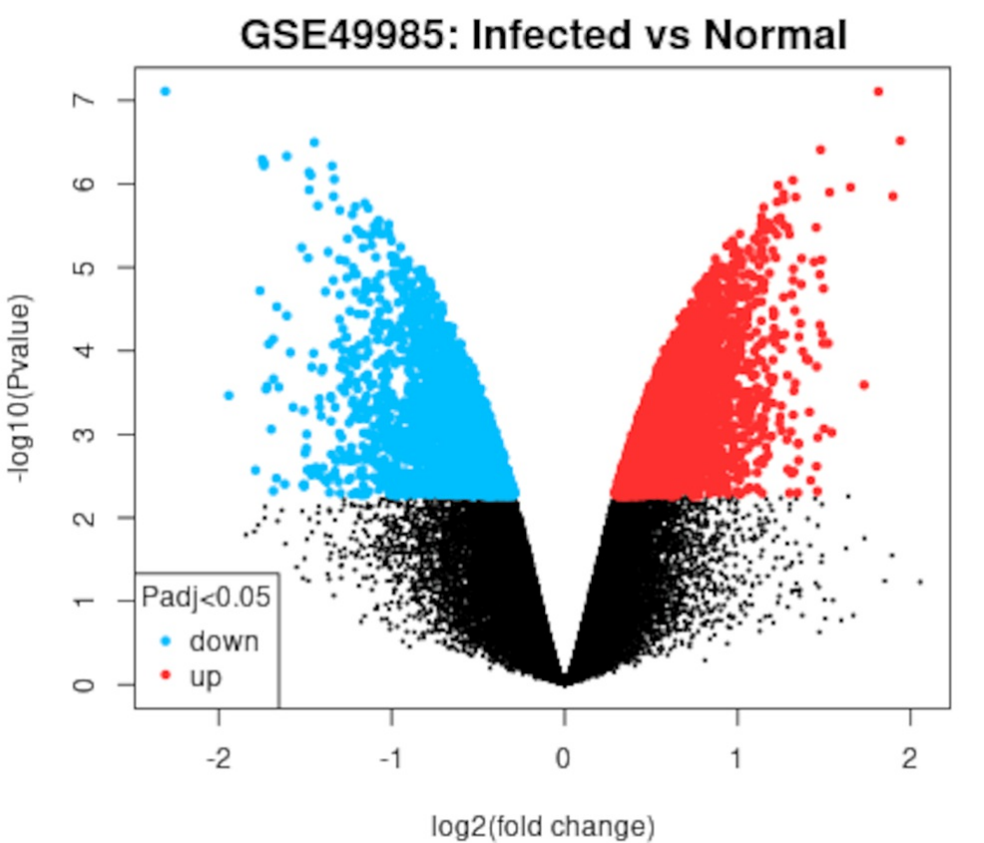
Volcano plot for the differentially expressed genes (DEGs) between the control and the infected samples. The red dots represent significantly upregulated genes, the blue dots show the significantly downregulated genes, and the black dots represent genes with no significant differential expression, as obtained from the Gene Expression Omnibus (GEO) database. (https://www.ncbi.nlm.nih.gov/geo/geo2r/).

Analysis of the GEO dataset GSE49985 with the default parameters of p < 0.05 and |log2FC ≥ 2| showed 48,821 DEGs between the control and the sample, of which 24,772 were upregulated and 24,048 were downregulated. Only one gene showed similar expression. A volcano plot showing the DEGs obtained from GEO database is displayed in Figure 1.

DEG identification

After the filtration of DEGs using the parameters of adjusted p < 0.05 and |log2FC ≥1.5|, there were 25 genes with highly significant differential expression, of which 21 genes were upregulated and four genes were downregulated, as presented in Table 1.

**Table 1:** The highly significant differentially expressed genes (DEGs) between the control and infected samples with adjusted p < 0.05 and log2FC ≥ 1.5 from the microarray dataset GSE49985.

Gene symbol	Adjusted p-value	Log_2_FC	Regulation
*CXCR4*	0.0032	-1.6520	Down
*SLC25A27*	0.0064	-1.5218	Down
*TMEM255A*	0.0100	-1.7301	Down
*ARPP19*	0.0188	-1.5432	Down
*ZNF440*	0.0019	2.3081	Up
*SMARCB1*	0.0030	1.6057	Up
*AP2M1*	0.0030	1.7483	Up
*HMGB1*	0.0030	1.7384	Up
*ERI3*	0.0039	1.5202	Up
*DDX3X*	0.0044	1.7607	Up
*CFL1*	0.0049	1.6636	Up
*TXNRD1*	0.0052	1.6060	Up
*RBM14*	0.0062	1.6845	Up
*MAT2A*	0.0064	1.7101	Up
*PPP2R1A*	0.0093	1.6830	Up
*C9orf16*	0.0103	1.6524	Up
*SUB1*	0.0115	1.9409	Up
*MGAT4B*	0.0134	1.5693	Up
*CNOT3*	0.0141	1.5080	Up
*LAMTOR1*	0.0180	1.6956	Up
*PABPC4*	0.0331	1.7867	Up
*HIF1A*	0.0378	1.6662	Up
*ACTB*	0.0413	1.6177	Up
*PHB2*	0.0429	1.5067	Up
*CTNNB1*	0.0463	1.6837	Up

PPI network construction

**Figure 2:**
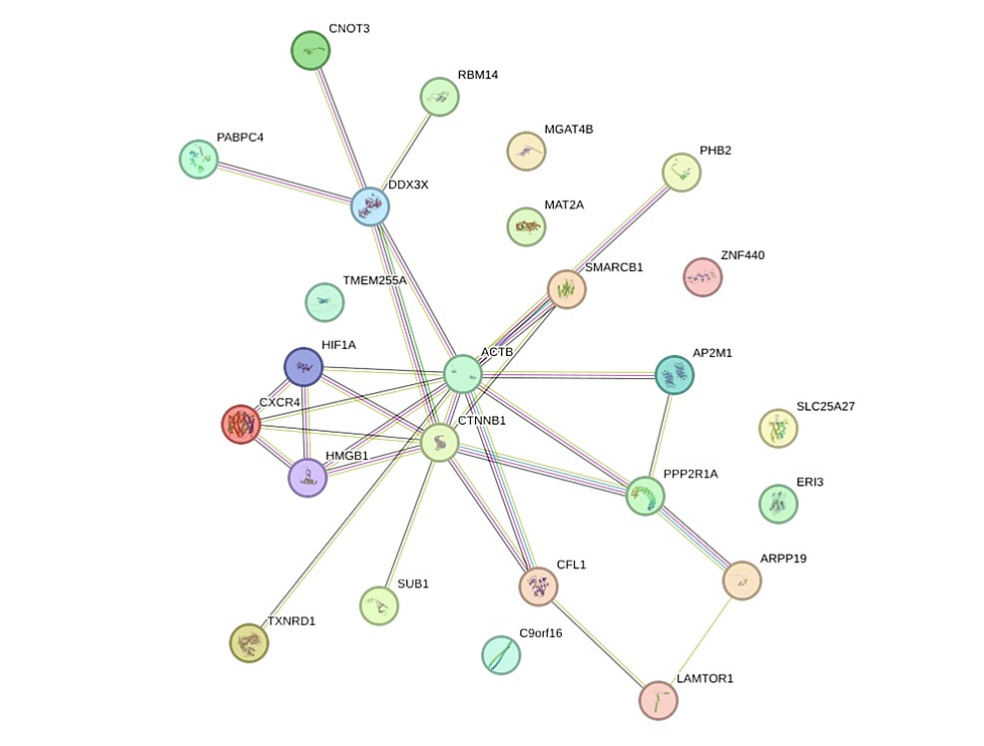
The protein-protein interaction (PPI) network done by Search Tool for the Retrieval of Interacting Genes/Proteins (STRING) database (http://string-db.org) for the 25 highly significant differentially expressed genes (DEGs). Colored circles indicate the assigned genes/proteins connected with lines to highlight the interactions between these proteins within the network.

Using the STRING database for PPI network analysis, the 25 highly significant DEGs were analyzed to identify the interactions of proteins produced by these genes. The results showed that there were 25 nodes with an average node degree of 2.32, edge number of 29, and an expected number of edges of 18. The average local clustering coefficient was 0.49 and the PPI enrichment p-value was 0.00988. The PPI generated by the STRING database is illustrated in Figure 2.

Constructing the PPI network by Cytoscape using the STRING plugin showed a network with 25 nodes and 29 edges; however, the average number of neighbors was 3.222 with a clustering coefficient of 0.347, which was slightly lower than the one generated by the STRING platform.

Hub genes and cluster identification

The PPI network was then analyzed using the CytoHubba plugin by Cytoscape to identify the hub genes, with *ACTB *and *CNNTB1 *genes showing the highest scores of 36 and 33, respectively, followed by *HIF1A*, *HMGB1*, *CXCR4*, *DDX3X*, *PPP2R1A*, *CFL1*, *ARPP19*, and *SMARCB1 *with scores of 24, 24, 24, 5, 5, 3, 2, and 2, respectively.

The top 10 hub genes network had 18 nodes and 29 edges with a 3.222 average number of neighbors and 0.347 as the clustering coefficient.

**Figure 3:**
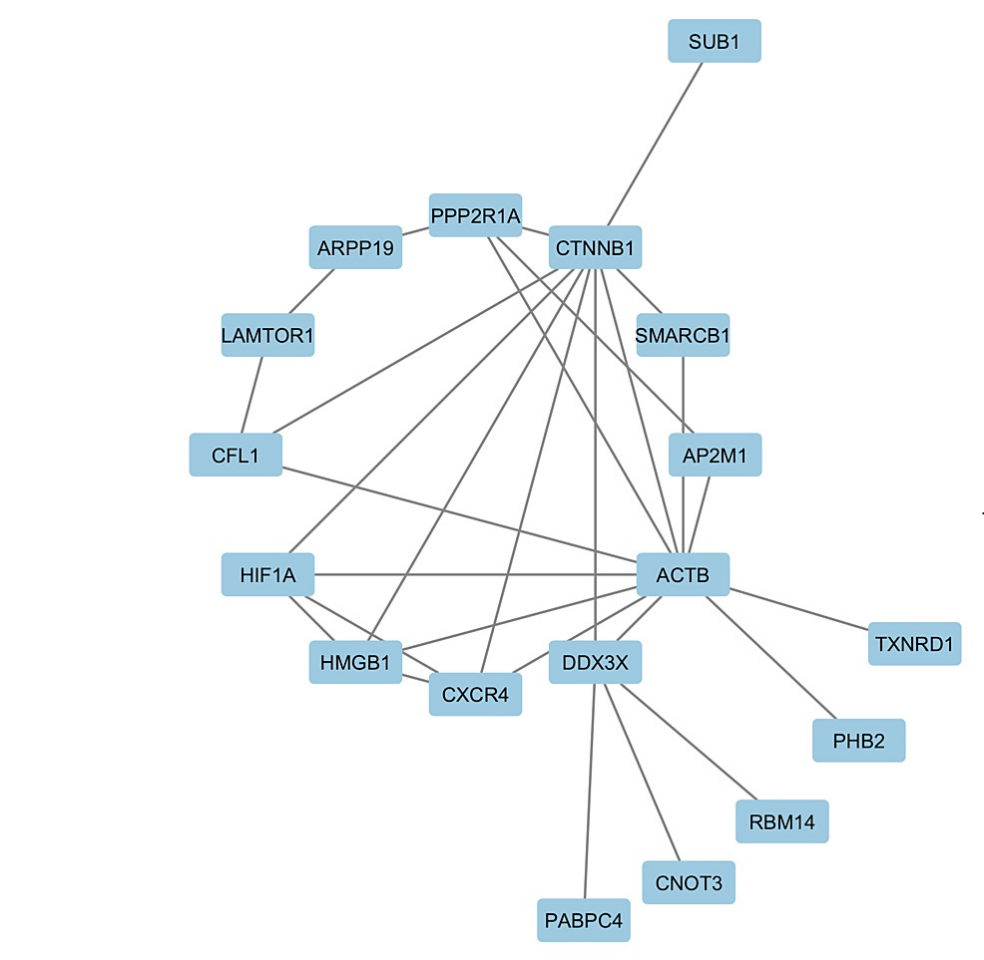
The constructed protein-protein interaction (PPI) network done by Cytoscape software (https://cytoscape.org/) using the Search Tool for the Retrieval of Interacting Genes/Proteins (STRING) plugin for the 25 highly significant differentially expressed genes (DEGs). Only genes involved in the protein interaction network are displayed.

**Figure 4:**
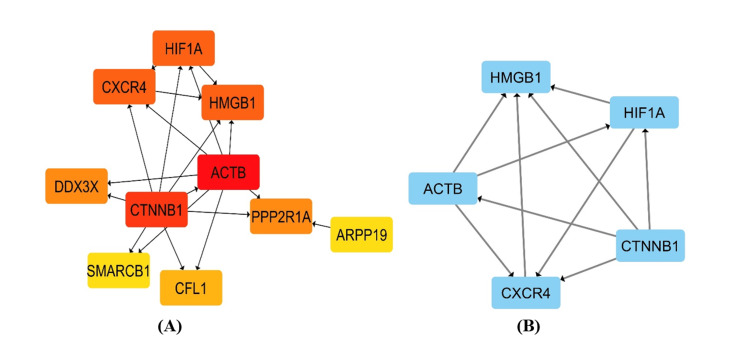
The visualization done by the Cytoscape software (https://cytoscape.org/). (A) The top 10 hub genes predicted by the CytoHubba plugin using the Maximum Clique Centrality (MCC) algorithm. The color shades indicate the ranking of gene interaction, with the red color representing genes with the highest rank, the yellow color showing genes with the lowest rank, and the orange shades displaying genes ranked in between. (B) The top cluster of the genes analyzed by the Molecular Complex Detection (MCODE) plugin on Cytoscape.

The MCODE plugin was employed to identify the top gene clusters involved in the network which showed one cluster whose score for density of nodes was 5, including 5 nodes and 10 edges with an average number of neighbors as 4 and clustering coefficient as 1. Five genes of the highest interactions in the PPI network were identified as follows: *ACTB*, *CNNTB1*, *HIF1A*, *HMGB1*, and *CXCR4*. Networks generated by Cytoscape are shown in Figure 3 and Figure 4.

Enrichment analysis using the KEGG pathway for the hub genes

**Figure 5:**
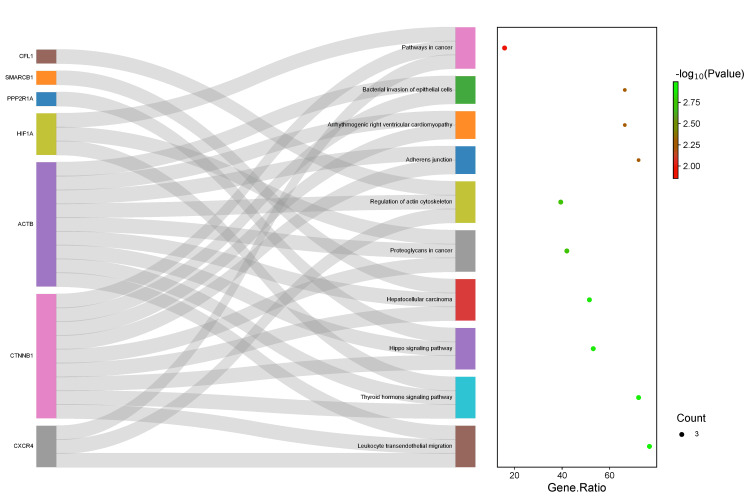
The Sankey plot visualized (http://www.bioinformatics.com.cn/) of the top 10 Kyoto Encyclopedia of Genes and Genomes (KEGG) pathways and the hub genes involved. The genes are shown on the left side and pathways are on shown the right side while the gene ratio is expressed by the p-value slope.

The 10 hub genes were analyzed by Enricher using the KEGG pathway to define the pathways at which these genes were involved. The results showed a total of 81 pathways, of which only 24 were significant pathways with adjusted p < 0.05. However, only the following top 10 pathways were obtained: leukocyte transendothelial migration, thyroid hormone signaling pathway, Hippo signaling pathway, hepatocellular carcinoma, proteoglycans in cancer, regulation of actin cytoskeleton, adherens junction, arrhythmogenic right ventricular cardiomyopathy, bacterial invasion of epithelial cells, and pathways in cancer. Following the enricher data analysis, a Sankey plot was constructed using http://www.bioinformatics.com.cn/ for these KEGG pathways to identify the relationship between the genes and the pathways. The Enrichr analysis results are demonstrated in Table 2 and Figure 5.

**Table 2:** The top 10 Kyoto Encyclopedia of Genes and Genomes (KEGG) pathways and the hub genes involved in these pathways with adjusted p < 0.05, as obtained from the Enrichr database (https://maayanlab.cloud/Enrichr/).

Number	KEGG pathway	Adjusted p-value	Genes involved
1	Leukocyte transendothelial migration	0.001018	*CXCR4*/*CTNNB1*/*ACTB*
2	Thyroid hormone signaling pathway	0.001018	*CTNNB1*/*HIF1A*/*ACTB*
3	Hippo signaling pathway	0.001355	*PPP2R1A*/*CTNNB1*/*ACTB*
4	Hepatocellular carcinoma	0.001355	*SMARCB1*/*CTNNB1*/*ACTB*
5	Proteoglycans in cancer	0.001956	*CTNNB1*/*HIF1A*/*ACTB*
6	Regulation of actin cytoskeleton	0.001956	*CFL1*/*CXCR4*/*ACTB*
7	Adherens junction	0.005808	*CTNNB1*/*ACTB*
8	Arrhythmogenic right ventricular cardiomyopathy	0.005808	*CTNNB1*/*ACTB*
9	Bacterial invasion of epithelial cells	0.005808	*CTNNB1*/*ACTB*
10	Pathways in cancer	0.014044	*CXCR4*/*CTNNB1*/*HIF1A*

**Figure 6:**
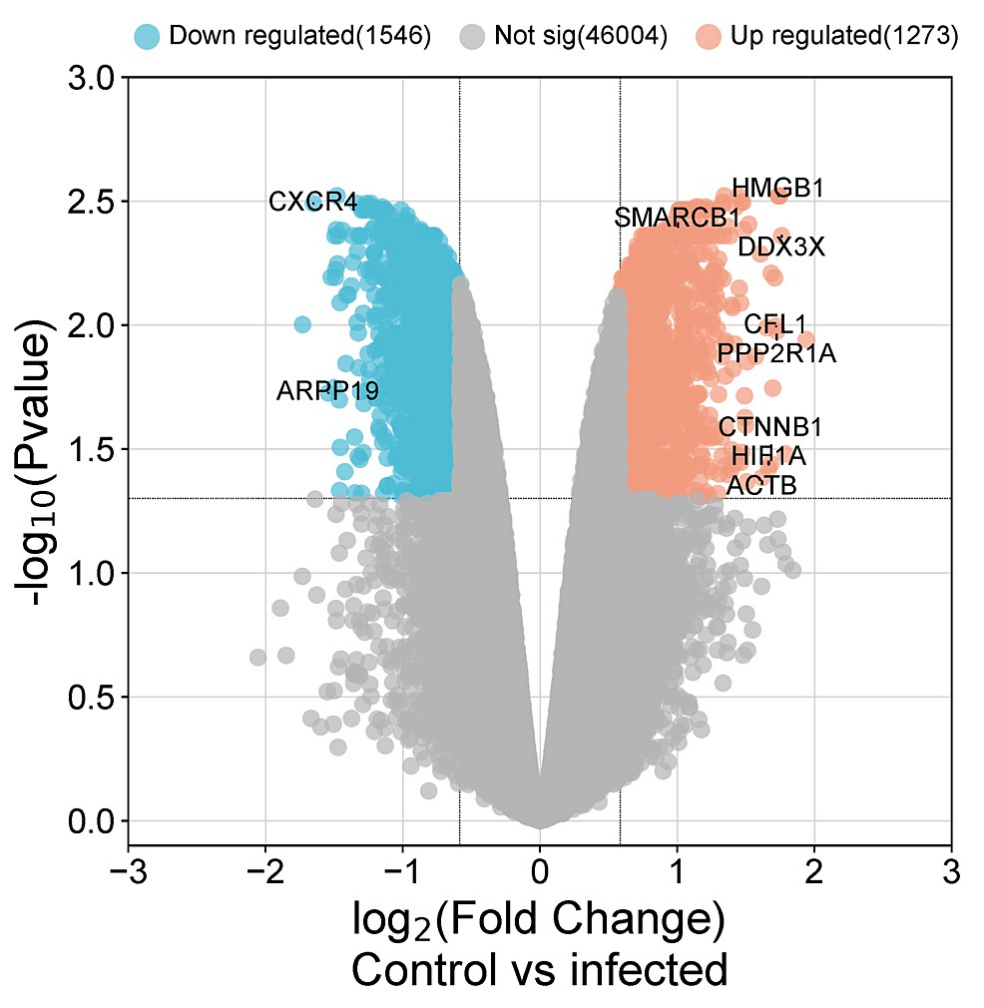
The reconstructed volcano plot of the hub genes marking their location within the differentially expressed genes (DEGs) in the microarray dataset displayed by (http://www.bioinformatics.com.cn/). Blue dots are downregulated genes, red dots are upregulated genes, and the non-significant genes are displayed in gray dots.

Accordingly, *ACTB* was involved in nine out of the 10 pathways making it the most important gene in the network, followed by *CTNNB1*,which was involved in eight pathways, *HIF1A* and *CXCR4*, which were involved in three signaling pathways, and *CFL1*, *PPP2R1A*, and *SMARCB1*, each involved in a single pathway. In addition, a reconstructed volcano plot of the hub genes revealed that *ACTB*, *CTNNB1*, *PPP2R1A*, *HIF1A*, *HMGB1*, *SMARCB1*, *DDX3X*, and *CFL1 *were significantly upregulated and *CXCR4 *and *ARPP19 *were significantly downregulated, as displayed in Figure 6.

The biological processes, cellular components, and molecular functions of these genes were also obtained as ontologies provided by Enrichr, and their results were visualized using GO enrichment from http://www.bioinformatics.com.cn/. These findings were crucial to understanding the nature of these designated hub genes to further comprehend their nature and connections.

**Figure 7:**
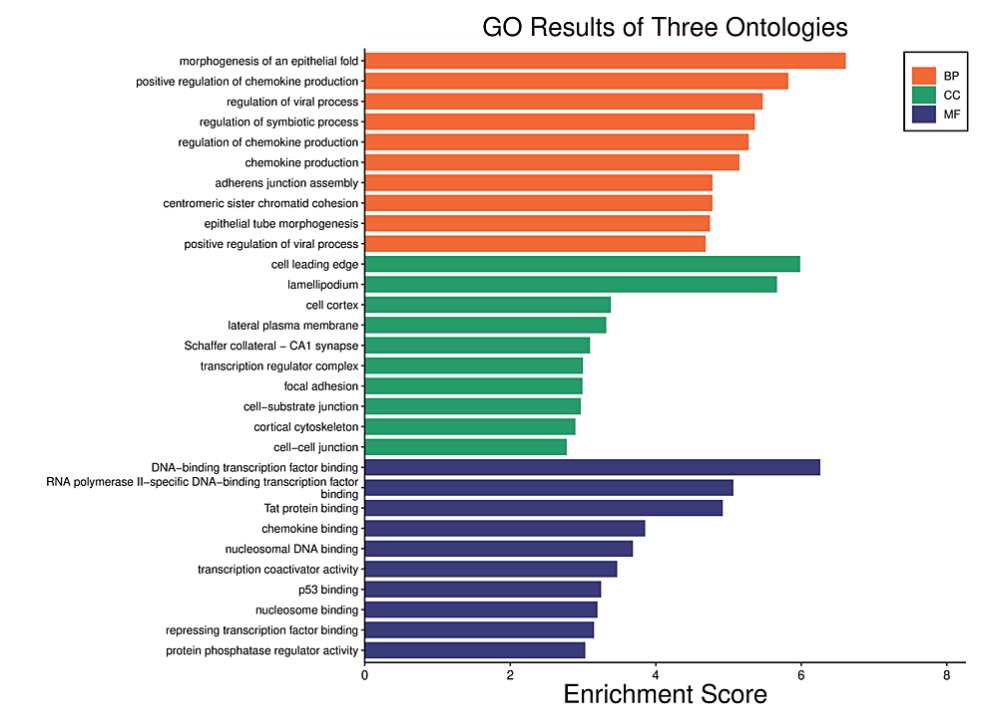
The Gene Ontology (GO) enrichment for the hub genes visualized by (http://www.bioinformatics.com.cn/) revealing the top 10 ranked pathways of BP, CC, and MF. BP = biological processes; CC = cellular components; MF = molecular functions

**Figure 8:**
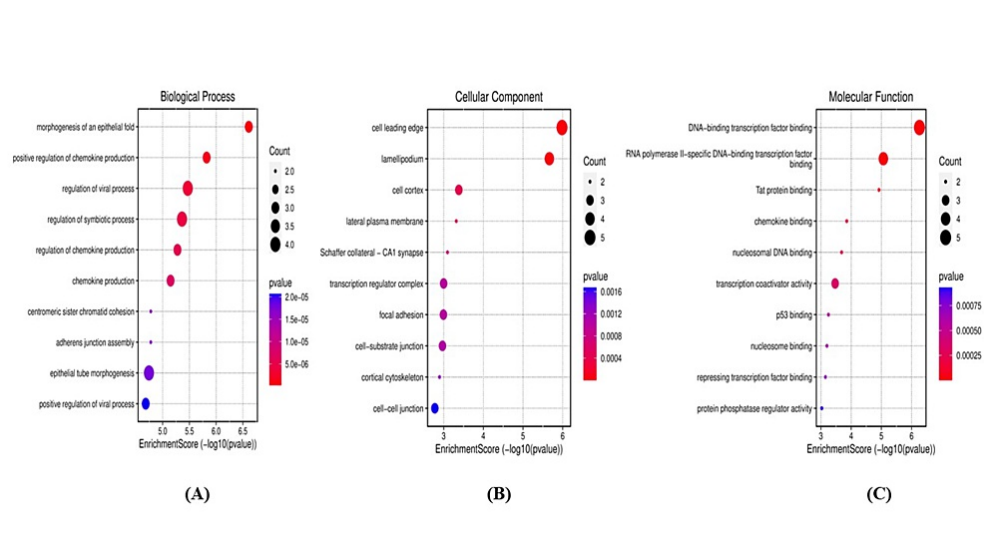
The top 10 Gene Ontology (GO) enrichment pathways of the hub genes visualized by (http://www.bioinformatics.com.cn/) and ranked according to p-values. (A) Represents biological processes. (B) Displays cellular components. (C) Represents pathways of molecular functions.

From the biological processes, only the top 10 ranked ontologies according to the p-value were taken and the same was applied to the cellular components and molecular functions. It was found that these hub genes play a role mainly on biological and molecular levels, significantly concerning synthesis, regulation, activation, and binding process as the CHIKV interacts with the immune cells, particularly dendritic cells and leukocytes. The obtained ontologies are displayed in Figure 7 and Figure 8.

## Discussion

The host genome and transcriptome are among the most important areas of research as they can provide insights into host susceptibility, transmission, disease severity, patient outcomes, and responses to vaccines and antiviral drugs. By studying the similarities and differences across infected host cells and comparing them to non-affected cells, vital information regarding intervention strategies and next-generation drug discovery could be extracted.

This study focused on identifying DEGs associated with CHIKV infection. Analysis of the microarray data of GSE49985 revealed a total of 25 DEGs, including 21 upregulated genes and four downregulated genes. This characterization of the DEGs as well as the hub genes plays an important role in understanding the molecular responses triggered by CHIKV infection and may reveal important mechanisms underlying its pathogenesis. The construction of the PPI network using the STRING platform and Cytoscape enabled the exploration of interactions between the DEGs. Further analysis with CytoHubba identified key hub genes in the network, particularly *ACTB *and *CTNNB1*, revealing extensive interactions and central roles in protein connectivity.

In this study, several pathways from the top 10 most significant KEGG pathways belonged to those involved in cellular signaling activated by viral infection. These cellular pathways are activated as part of the host’s defense mechanisms or in response to the virus’s attempt to hijack cellular machinery for replication and survival.

One of these pathways and the most significant one in this study is leukocyte transendothelial migration which is a mechanism used by leukocytes to cross the endothelium lining of the vasculature initiated by intracellular signaling that controls leukocyte adhesion, spreading, and motility. This pathway is also involved in inflammatory mechanisms initiated due to viral genome integration and protein synthesis which leads to the activation and release of inflammatory and pro-inflammatory mediators such as interferons and interleukins, as well as antiviral signaling pathways to enhance viral recognition and destruction. Viruses can manipulate the endothelium and transendothelial migration to their advantage, promoting the spread of infection. This has been observed in many viral infections, including human immunodeficiency virus (HIV), measles, respiratory syncytial virus (RSV), and cytomegalovirus (CMV) [12].

Other pathways involved in viral infection and belonging to the most significant KEGG pathways in this study were the regulation of actin cytoskeleton and adherens junction, which play a pivotal role in viral infection processes.

Modulation of cellular signaling involved in cytoskeletal dynamics by the virus can cause cytoskeletal rearrangements, aiding viral entry and transport within the cell. This pathway was particularly noticed in insect viruses [13] such as CHIKV. Moreover, it was found that many viruses, for example, HIV, hepatitis C virus (HCV), and human papillomavirus (HPV), can interact with various types of cell junctions (tight, gap, and adherens) through different mechanisms, thereby affecting cellular integrity and migration and facilitating viral invasion and replication [14].

The second most significant KEGG pathway was the thyroid hormone signaling pathway. The role of thyroid hormones in viral infection has been the subject of extensive research; for example, in herpes simplex virus (HSV) infection, clinical observations and in vivo experiments suggest that thyroid hormone T3 is involved in suppressing the virus replication during reactivation [15]. This finding could suggest a significant interaction between thyroid hormones and CHIKV infection.

The Hippo pathway plays a critical role in cell proliferation, organ development, tumorigenesis, and regulation of adaptive immunity and could be manipulated by viruses to promote their replication and pathogenesis. For example, viruses such as hepatitis B virus (HBV), Epstein-Barr virus (EBV), HCV, and HPV have been found to regulate the Hippo pathway during viral infection, which could also contribute to their carcinogenic functions [16].

Three of the top 10 significant pathways were related to cancer, i.e., hepatocellular carcinoma, pathways in cancer, and proteoglycans in cancer.

In general, hepatic involvement during CHIKV infection was usually observed only during the first two weeks of illness and resolved spontaneously. No hepatitis, liver damage, or failure has been observed in patients infected with CHIKV [17].

It is well-known that viruses can initiate the same cellular signaling pathways as cancer. Tumor viruses have been instrumental in the discovery and understanding of cancer biology. This similarity is due to many factors, for instance, both virus infections and the development of cancer often involve long latent periods between the initial infection or transformation and the appearance of clinical symptoms or tumors. In both host factors, genetic susceptibility and immune response play a crucial role in determining susceptibility to viral infections and cancer. Moreover, viral strains may differ in biologic properties similar to the heterogeneity observed in different types of tumors [18].

Regarding proteoglycans in the cancer pathway, proteoglycans, particularly heparan sulfate proteoglycans, have been shown to play an important role in the attachment and uptake of the virus, thereby being directly involved in viral infectivity and cellular invasion, particularly in SARS-CoV-2 infection [19].

The KEGG pathway arrhythmogenic right ventricular cardiomyopathy was an interesting finding as cardiovascular manifestations with CHIKV infection have been reported in various countries, especially in Asia and the Americas. Some of the reported cardiac symptoms include chest pain, fatigue, dyspnea, edema, vagal symptoms, hypotension, tachycardia, tachypnea, shock, circulatory collapse, edema, Raynaud phenomenon, arrhythmias, murmurs, myocarditis, dilated cardiomyopathy, congestive insufficiency, and heart failure [20].

Regarding the bacterial invasion of the epithelial cell pathway, and according to the literature, bacterial co-infection with CHIKV has been documented once in Colombia with dengue and leptospira [21]. In general, there are similarities between the bacterial and viral invasion of epithelia as both have evolved strategies to target, cross, or disrupt intercellular junctions and components of epithelial barriers. It has been well documented that many respiratory viruses such as RSV and influenza enhance the adhesion of bacterial pathogens such as non-typeable *Haemophilus influenzae* and *Streptococcus pneumoniae* to respiratory epithelium in a virus species-cell type-dependent manner [22]. This finding may suggest that CHIKV may play a role in the modulation of bacterial epithelial invasion that needs extensive research to be validated.

The hub genes identified in this study represent the genes with the most significant expression. The major key players in the PPI network were genes involved in the regulation of various cellular signaling pathways that show complex intertwined with each other. As mentioned earlier, these pathways match those performed by the host’s body in response to viral infection such as cytoskeleton dynamics, initiation of innate immune response and inflammation, and oxidative stress responses such as apoptosis. In addition, many of these genes were proposed as potential therapeutic targets for various viral infections.

The top five genes that formed the only cluster with highly interconnected regions in the network were *ACTB*, *CTNNB1*, *CXCR4*, *HIFIA*, and *HMGB1*.

*ACTB* (actin b) is a gene that encodes one of six highly conserved actin proteins involved in cell motility, structure, integrity, and intercellular signaling. It has been reported that ACTB protein can increase the replication efficiency of HIV and can be a potential biomarker in dengue virus infection [23].

The *CTNNB1* gene encodes β-catenin protein which is part of a complex of proteins that constitute adherens junctions. It also anchors the actin cytoskeleton and may be responsible for transmitting the contact inhibition signal that stops cell division once the epithelial sheet is formed [24]. β-catenin is an integral part of the canonical Wnt signaling pathway which is responsible for cell migration and has been found to be modulated by many viruses such as HIV, HCV, and influenza to enhance viral replication [25].

The *CXCR4* gene encodes the chemokine receptor CXCR4 which, along with its ligand CXCL12, plays a crucial role in various physiological processes, including cell migration, survival, and proliferation. It has a prominent function in orchestrating innate and adaptive immune responses and regulating leukocyte trafficking and distribution [26].

*CXCR4* was shown to be exploited by diverse viral pathogens for their survival and proliferation, ultimately affecting cell trafficking, immune responses, and antigen presentation. Viruses such as HIV, EBV, CMV, HPV, poxviruses, and human herpes virus have been found to decrease CXCR4-CXCL12 expression and signaling, thereby directing cells toward other chemokine signals, or preventing virus-infected cells from entering lymphoid organs to reduce the likelihood of antigen presentation and eliciting an antiviral immune response [27].

The *HMGB1* (high mobility group box 1) gene encodes a protein considered a critical proinflammatory cytokine that exacerbates inflammatory responses and disease severity and is implicated in the regulation of diverse types of cell death, such as apoptosis, necrosis, and pyroptosis, which are associated with virus infection-induced HMGB1 release. Studies have revealed that *HMGB1 *plays multifaceted roles in viral replication cycles, thereby either promoting or restricting viral infection in a manner dependent on the viral strain and cellular context. Intracellular *HMGB1 *has been found to positively regulate viral genes and facilitate virus replication by binding to viral proteins or genomic RNA, as seen in influenza A virus and HCV, or by the formation of viral enhanceosomes [28].

HIF1A (hypoxia-inducible factor 1) is a transcription factor that plays a critical role during the cellular response to hypoxia. There are some mechanisms by which different viruses modulate the expression and stabilization of HIF1A. Viruses such as HBV, EBV, HCV, HIV, and Influenza virus, enhance HIF1A expression which can lead to infected cell adaptation to hypoxic conditions promoting viral replication and survival. In addition, this can lead to activation of antiviral mechanisms and release of inflammatory cytokines [29].

According to our findings, *ACTB*, *CTNNB1*, *HMGB1*, and *HIF1A* genes were significantly upregulated in the infected CHIKV cells while *CXCR4* was significantly downregulated, suggesting that CHIKV could utilize these genes and the pathways involved to enhance its invasion, entry, and spread to the host cells as well as to evade viral recognition and removal by host cells.

Studies have proven that CHIKV employs various strategies through a complex series of mechanisms to establish infection, replicate efficiently, and evade the host’s immune responses, particularly in the initial stages of infection. The most prominent mechanisms are (1) immune evasion: when the virus utilizes various non-structural proteins (nsPs) to evade the host’s immune system. These proteins play a role in interrupting interferon signaling which is crucial for controlling viral infection and suppressing innate immune responses and antiviral signaling which helps the virus to escape host defense. (2) Modulation of host cell processes: when CHIKV utilizes the host’s machinery for its advantage by altering gene transcription and modifying cellular processes to create a favorable environment for viral replication [30].

While this study provides valuable insights into the molecular mechanisms of CHIKV infection, it is not without limitations. First, the study relied on publicly available datasets, which may contain inherent biases or variations in data quality. Additionally, the analysis was conducted in a bioinformatics context, and the findings should be validated through experimental studies. Furthermore, the study focused on one specific dataset and should be complemented by broader investigations to capture the full spectrum of CHIKV infection dynamics.

## Conclusions

Transcriptome analysis by RNA-based techniques to study changes in host transcription along with bioinformatics analysis are powerful tools that can uncover correlations between specific transcripts and their influence on host susceptibility, viral lifecycle, immune response, patient outcomes, intervention strategies, and potential infection biomarkers. Integrative bioinformatics analysis allowed us to identify important DEGs and decipher their involvement in specific biological pathways during CHIKV infection. Hub genes, especially *ACTB* and *CTNNB1* along with *CXCR4*, *HMGB1*, and *HIF1A*, showed significant connectivity and involvement in multiple signaling pathways, providing insights into their possible role in the biological and molecular process of the infection and may reveal new therapeutic targets or diagnostic markers.

The results reflect the role of understanding that the identified signaling pathways and hub genes in CHIKV infection provide the basis for targeted studies. Further investigation of the impact of these signaling pathways on viral pathogenesis, immune responses, and cellular function may facilitate the development of new antiviral strategies and diagnostic tools. Additional studies are required to focus on experimental validation and the use of in vitro or in vivo models to confirm the functional relevance of the identified genes and pathways in CHIKV infection. Moreover, the study of potential drug targets related to these pathways may aid in the development of targeted therapies to alleviate CHIKV-related morbidity.
